# Pharmacy Benefit Manager Pricing and Spread Pricing for High-Utilization Generic Drugs

**DOI:** 10.1001/jamahealthforum.2023.3660

**Published:** 2023-10-20

**Authors:** T. Joseph Mattingly, Kenechukwu C. Ben-Umeh, Ge Bai, Gerard F. Anderson

**Affiliations:** 1 Department of Pharmacotherapy, University of Utah College of Pharmacy, Salt Lake City; 2 Johns Hopkins Carey Business School, Baltimore, Maryland; 3 Johns Hopkins Bloomberg School of Public Health, Baltimore, Maryland

## Abstract

This cross-sectional study uses Medicare Part D claims for high-utilization generic drugs to analyze gross profits accumulated by pharmacy benefit managers, pharmacies, wholesalers, and manufacturers in the pharmaceutical supply chain.

## Introduction

The practice by pharmacy benefit managers (PBMs) of spread pricing—charging the client (ie, insurer) a higher amount than is reimbursed to the pharmacy—has garnered substantial attention from policymakers. The bipartisan proposals in the PBM Transparency Act^1^ and PBM Reform Act^2^ and numerous state laws^3^ prohibit spread pricing. However, others in the pharmaceutical supply chain, such as pharmacies and wholesalers, also rely on spread pricing to earn profits. The gross profit for each entity in the supply chain remains unexplored in the literature. Using Medicare Part D data, this study analyzed entity-level gross profit in the pharmaceutical supply chain for high-utilization generic drugs.

## Methods

We conducted a cross-sectional study of generic drugs covered by Medicare Part D with more than 2 manufacturers, accounting for more than $100 million in total Part D spending and impacting more than 1 million Medicare beneficiaries. The Medicare Part D Dashboard was used to determine total spending, units dispensed, claims, and beneficiaries for these drugs in 2021.^4^ We followed the STROBE reporting guideline. Approval and informed consent were waived per the University of Utah institutional review board because we did not include human participants.

Pharmacy revenue was estimated by using the Federal Upper Payment Limit (FUL) for each drug in 2021.^5^ The FUL is a statutorily determined price for generic drugs with multiple competitors and approximates maximum allowable price limits paid by PBMs.^5^ Wholesaler revenue was estimated using National Average Drug Acquisition Cost (NADAC) in 2021,^6^ which is based on a national survey of a random sample of pharmacies from all states estimating the price pharmacies pay to acquire drugs. Manufacturer revenue was estimated using average manufacturer price (AMP) reported in 2021. Therefore, PBM gross profit was estimated as Medicare Part D spending minus pharmacy revenue (FUL); pharmacy gross profit equals pharmacy revenue (FUL) minus wholesaler revenue (NADAC) and wholesaler gross profit equals wholesaler revenue (NADAC) minus manufacturer revenue (AMP). The remainder is manufacturer revenue. Manufacturer gross profit was not estimated due to lack of information on ingredient cost and other manufacturing expenses. Spread pricing was calculated at claim level (total spending divided by total claims). Analyses used Microsoft Excel, version 16.

## Results

In all, 45 drugs met inclusion criteria (Table 1). In 2021, Medicare Part D spent $11.8 billion for 690 million claims (mean, $22.50 per claim; 95% CI, $18.28-$26.72 per claim), representing 5.5% of all Part D spending ($216 billion) in 2021. The $22.50 was distributed as follows: $9.18 (40.8%) represents PBM gross profit; $3.87 (17.2%), pharmacy gross profit; $2.71 (12.0%), wholesaler gross profit; and $6.73 (29.9%), manufacturer revenue. Total intermediary gross profit (PBM, wholesaler, and pharmacy) ranged from −12.3% to 88.6% of Medicare Part D spending. Table 2 describes 2 case-study drugs with key sample data extracted.

**Table 1.** Medicare Part D Spending and Intermediary Gross Profit for High-Utilization Generic Drugs in 2021

Generic drug	Total spending, $	Total claims, No.	Spending per claim, $	Intermediary gross profit	Proportion of spending, %
Ezetimibe	285 698 641	5 008 626	57.04	47.56	83.4
Clobetasol propionate	118 407 665	1 875 448	63.14	43.55	69.0
Celecoxib	198 447 520	3 965 182	50.05	38.34	76.6
Valacyclovir HCl	112 298 507	2 540 155	44.21	32.51	73.5
Pregabalin	307 562 067	5 638 407	54.55	30.51	55.9
Duloxetine HCl	414 557 638	11 009 950	37.65	29.08	77.2
Baclofen	140 461 448	4 830 105	29.08	25.03	86.1
Rosuvastatin calcium	564 466 206	18 199 685	31.02	24.76	79.8
Diclofenac sodium	258 978 903	8 379 385	30.91	24.73	80.0
Potassium chloride	433 439 766	14 210 545	30.50	23.98	78.6
Doxycycline hyclate	108 947 882	4 328 095	25.17	22.31	88.6
Losartan and hydrochlorothiazide	122 708 535	4 675 200	26.25	21.56	82.1
Bupropion HCl	226 430 273	5 275 658	42.92	19.86	46.3
Mirtazapine	167 528 216	7 311 127	22.91	18.64	81.4
Oxycodone HCl and acetaminophen^a^	240 010 057	8 667 327	27.69	17.03	61.5
Metoprolol succinate	587 427 283	26 215 995	22.41	17.00	75.8
Finasteride	122 402 257	6 476 445	18.90	15.66	82.8
Buspirone HCl	113 534 416	5 976 927	19.00	15.00	78.9
Quetiapine fumarate	202 983 398	8 475 019	23.95	14.79	61.7
Pravastatin sodium	188 695 359	10 559 760	17.87	14.75	82.5
Pantoprazole sodium	375 326 098	20 999 275	17.87	14.72	82.3
Gabapentin	622 195 038	32 996 186	18.86	12.06	64.0
Montelukast sodium	163 129 688	11 318 679	14.41	11.71	81.3
Levothyroxine sodium	728 257 030	38 443 070	18.94	11.54	60.9
Hydrocodone and acetaminophen	458 937 357	21 200 893	21.65	11.47	53.0
Omeprazole	439 420 730	28 270 377	15.54	11.24	72.3
Escitalopram oxalate	152 321 907	10 603 618	14.37	11.19	77.9
Allopurinol	135 198 663	8 988 289	15.04	10.47	69.6
Famotidine	200 951 499	10 633 240	18.90	10.40	55.0
Donepezil HCl	110 126 466	7 374 685	14.93	9.99	66.9
Atorvastatin calcium	836 195 879	61 100 574	13.69	9.42	68.9
Carvedilol	162 600 636	14 728 517	11.04	9.19	83.2
Losartan potassium	392 886 345	29 424 165	13.35	8.63	64.6
Clopidogrel bisulfate	158 314 799	12 930 126	12.24	8.59	70.2
Fluoxetine HCl	100 275 333	6 920 559	14.49	8.41	58.0
Alprazolam	118 722 365	11 930 537	9.95	7.91	79.5
Sertraline HCl	159 175 693	14 268 296	11.16	7.55	67.7
Trazodone HCl	188 771 115	15 158 326	12.45	6.75	54.2
Simvastatin	163 875 183	18 748 527	8.74	6.71	76.7
Metoprolol tartrate	137 111 040	17 454 387	7.86	6.47	82.4
Amlodipine besylate	310 021 008	44 200 206	7.01	6.07	86.6
Lisinopril	259 836 409	36 125 959	7.19	5.78	80.4
Metformin HCl	181 651 525	24 245 215	7.49	5.18	69.2
Furosemide	140 004 950	23 438 082	5.97	4.48	75.0
Oxycodone HCl^a^	180 530 233	7 486 820	24.11	–2.96	−12.3

Abbreviation: HCl, hydrochloride.

^a^ Immediate-release oxycodone HCl is available in 5 different tablet strengths and as a capsule with wide variation in unit-level pricing. The average manufacturer price reported was higher than the reported Medicare Part D spending per unit, potentially reflecting increased utilization of the lowest-price strength and dose form.

**Table 2.** Product-Specific Case Studies for Donepezil Hydrochloride and Oxycodone and Acetaminophen Using 2021 Data

Variable	Donepezil hydrochloride	Oxycodone and acetaminophen	Sources and notes
Medicare Part D spending, $	110 126 466	240 010 057	2021 Medicare Part D Dashboard figures are based on gross drug costs including all spending on claims, plan, and beneficiary payments. These figures do not include manufacturers’ rebates or other price concessions that CMS is prohibited from disclosing.
Total prescription claims, No.	7 374 685	8 667 327	
Part D spending per prescription, $	14.93	27.69	
Total dose units, No.	362 157 114	680 757 903	
FUL per unit, $	0.2044	0.2376	The FUL is a statutorily determined price for generic drugs that have multiple competitors. These data are available per dose unit, and the average payment for the prescription can be calculated based on the mean units in each claim.
FUL per prescription, $	10.04	18.66	
NADAC per unit, $	0.1673	0.1908	NADAC data provide weekly reference data on drug prices at the product unit level, and the average payment for the prescription can be calculated based on the average units in each claim. These prices are based on a national survey of a random sample of pharmacies from all states estimating the price pharmacies pay to acquire drugs.
NADAC per prescription, $	8.22	14.99	
AMP per unit, $	0.1006	0.1358	The AMP is reported by manufacturers to CMS quarterly and is available at the product unit level. The average payment for the prescription can be calculated based on the average units in each claim. The AMP may not include prompt pay discounts to wholesalers and excludes direct sales to nursing homes.
AMP per prescription, $	4.94	10.67	
**PBM financials**			
Revenue per prescription, $	14.93	27.69	We estimated PBM revenue from Medicare Part D spending.
COGS per prescription, $	10.04	18.66	PBM COGS was estimated based on the prescription-level FUL as a proxy for the payment to the pharmacy.
Gross profit per prescription, $	4.89	9.03	The difference represents an estimated gross profit amount for the PBM that may factor into administrative fees, premiums, etc.
**Pharmacy financials**			
Revenue per prescription, $	10.04	18.66	We estimated pharmacy revenue using the prescription-level FUL as a proxy for payment from the PBM, as most multisourced generics may be on PBM MAC lists.
COGS per prescription, $	8.22	14.99	Pharmacy COGS was estimated based on the prescription-level NADAC survey reported by a national sample of pharmacies.
Gross profit per prescription, $	1.82	3.67	The difference represents an estimated gross profit amount for the pharmacy prior to paying for operating expenses.
**Wholesaler financials**			
Revenue per prescription, $	8.22	14.99	We estimated the wholesaler revenue based on the prescription-level NADAC paid by the pharmacy to acquire the product.
Cost of goods sold per prescription, $	4.94	10.67	Wholesaler COGS was estimated from the manufacturer-reported AMP.
Gross profit per prescription, $	3.82	4.32	The difference represents an estimated gross profit amount for the wholesaler prior to paying for operating expenses.
**Manufacturer financials**			
Revenue per prescription, $	4.94	10.67	Manufacturer revenue was estimated using prescription-level AMP.

Abbreviations: AMP, average manufacturer price; CMS, Centers for Medicare & Medicaid Services; COGS, cost of goods sold; FUL, federal upper payment limit; MAC, maximum allowable cost; NADAC, National Average Drug Acquisition Cost; PBM, pharmacy benefit manager.

## Discussion

In 2021, all but 29.9% of Medicare Part D dollars spent on 45 high-utilization generic drugs went to intermediary gross profit. This study was limited by the lack of information on direct and indirect remuneration offered by manufacturers or pharmacies. Without data on operating expenses, we were unable to estimate net profits generated. Additionally, without access to the proprietary agreements between the Centers for Medicare & Medicaid Services (CMS) and PBMs or plan sponsors, we were unable to determine what proportion of gross profits earned on these generic drug transactions was returned to CMS or used to reduce premiums. To the extent that the FUL exceeds actual pharmacy revenue for certain drugs in Medicare Part D, our estimated PBM spread pricing may be underestimated.

Policy efforts prohibiting spread pricing practices of PBMs may lower claim-level revenue retained by PBMs for generic drugs. However, absent sufficient market competition, PBMs may raise administrative fees to sustain revenue. Therefore, it remains unclear how much spread pricing reform focused on PBMs alone would lower drug spending or strengthen the generic pharmaceutical supply chain.

## Notes

Supplement.
Data Sharing Statement
