# Supplementary material for: Pharmacy Benefit Manager Pricing and Spread Pricing for High-Utilization Generic Drugs
Source: JAMA Health Forum. 2023 Oct 20;4(10):e233660. doi: 10.1001/jamahealthforum.2023.3660 (PMC10589804; doi:10.1001/jamahealthforum.2023.3660)
Supplement: Supplement. — Data Sharing Statement [file jamahealthforum-e233660-s001.pdf]

Data Sharing Statement

Mattingly, II. Pharmacy Benefit Manager Pricing and Spread Pricing for High-Utilization Generic Drugs. *JAMA Health Forum*. Published October 20, 2023.
doi:10.1001/jamahealthforum.2023.3660

Data
Data available: No

Additional Information
Explanation for why data not available: All data required to verify the findings are included in the submission.
